# The Role of IgLON Cell Adhesion Molecules in Neurodegenerative Diseases

**DOI:** 10.3390/genes14101886

**Published:** 2023-09-28

**Authors:** Marco Salluzzo, Clara Vianello, Sandra Abdullatef, Roberto Rimondini, Giovanni Piccoli, Lucia Carboni

**Affiliations:** 1 Department of Pharmacy and Biotechnology, Alma Mater Studiorum University of Bologna, 40126 Bologna, Italy; marco.salluzzo@unibo.it; 2 Department of Medical and Surgical Sciences, Alma Mater Studiorum University of Bologna, 40126 Bologna, Italy; clara.vianello2@unibo.it (C.V.); roberto.rimondini@unibo.it (R.R.); 3 Department of Cellular, Computational and Integrative Biology, University of Trento, 38123 Trento, Italy; sandra.abdullatef@unitn.it (S.A.); giovanni.piccoli@unitn.it (G.P.)

**Keywords:** IgLON5, LSAMP, NEGR1, NEUROTRIMIN, OPCML, Alzheimer’s disease, Huntington’s disease, Synucleinopathies, Niemann-Pick disease, psychiatric disorders

## Abstract

In the brain, cell adhesion molecules (CAMs) are critical for neurite outgrowth, axonal fasciculation, neuronal survival and migration, and synapse formation and maintenance. Among CAMs, the IgLON family comprises five members: Opioid Binding Protein/Cell Adhesion Molecule Like (OPCML or OBCAM), Limbic System Associated Membrane Protein (LSAMP), neurotrimin (NTM), Neuronal Growth Regulator 1 (NEGR1), and IgLON5. IgLONs exhibit three N-terminal C2 immunoglobulin domains; several glycosylation sites; and a glycosylphosphatidylinositol anchoring to the membrane. Interactions as homo- or heterodimers in *cis* and in *trans*, as well as binding to other molecules, appear critical for their functions. Shedding by metalloproteases generates soluble factors interacting with cellular receptors and activating signal transduction. The aim of this review was to analyse the available data implicating a role for IgLONs in neuropsychiatric disorders. Starting from the identification of a pathological role for antibodies against IgLON5 in an autoimmune neurodegenerative disease with a poorly understood mechanism of action, accumulating evidence links IgLONs to neuropsychiatric disorders, albeit with still undefined mechanisms which will require future thorough investigations.

## 1. Introduction

Cell adhesion molecules (CAMs) placed on the cell surface perform critical functions in a number of biological processes requiring contact between cells or with the extracellular matrix, such as cell recognition, adhesion, migration, and differentiation. The principal CAM groups are integrins, selectins, cadherins, and the immunoglobulin superfamily. In the developing brain, CAMs are crucial for the correct assembly of synaptic connections as well as the interactions with supporting glial cells. In the mature brain, complex functions depend on the correct performance of CAMs in establishing contacts between neuronal bodies, axonal interactions with myelinating glial cells, axon fasciculation, and connections to non-nervous cells [1,2]. Integrins are type I transmembrane proteins consisting of a large multidomain extracellular portion, a single-pass transmembrane region, and a short cytoplasmic component. The integrin family encompasses heterodimers of α and β subunits, which can combine to form several different integrins exhibiting overlapping but non-redundant functions, with specific ligand and signalling preferences depending on the α and β subunit combinations [3,4]. Selectins consist of an N-terminal carbohydrate-recognition domain allowing for the binding to glycoconjugates, an epidermal growth factor-like domain, a series of short consensus repeats, a transmembrane region, and a short C-terminal intracellular tail. Three selectin family members exist, P-selectin, L-selectin, and E-selectin, expressed in platelet-endothelial cells, leukocytes, and endothelial cells, respectively [5,6]. In the brain, selectins expressed by endothelial cells are involved in inflammatory responses, in damage after ischemic events, and in autoimmune diseases [6,7,8]. Cadherins are a large number of calcium-dependent adhesion proteins. Their structure comprises a calcium-binding extracellular domain consisting of several cadherin repeats of about 100 amino acids, a transmembrane domain, and a cytoplasmic domain which interacts with signalling molecules. Cadherins can form lateral dimers (cis-dimers) as well as trans-dimers with cadherins expressed by other cells [9,10].

**Figure 1:**
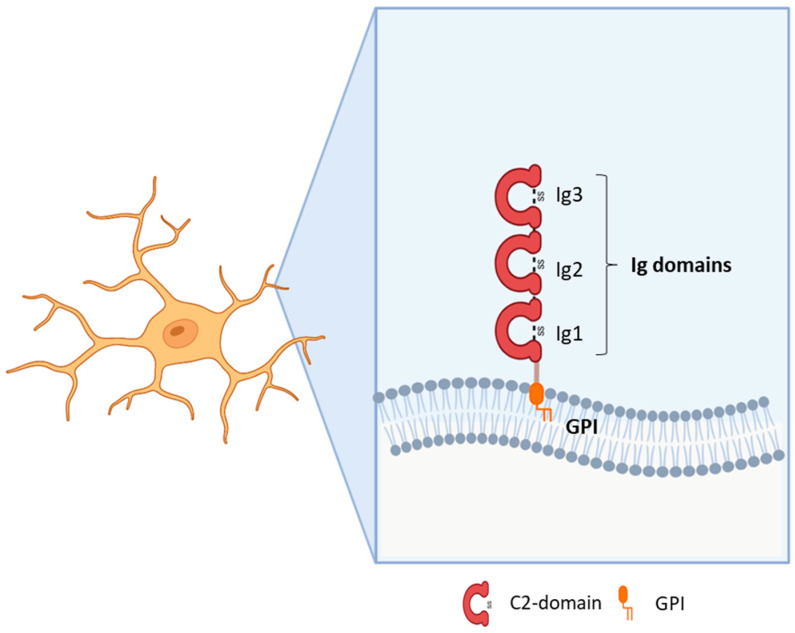
Structure of IgLON family members.

Immunoglobulin (Ig)-like molecules are an ancient and diverse family of proteins performing a variety of functions, such as immune and signalling molecules and CAMs. Four different subtypes of Ig-like domains exist which are named constant 1, constant 2, variable, and intermediate (C1, C2, V, and I) for their resemblance to immunoglobulin domains. The presence of at least one Ig-like domain is a requirement of this class [11,12]. Generally, the core of all Ig domains encompasses two β-sheets facing each other and an intra-chain disulfide bridge which provides stability to the structure [13]. In addition, variable numbers of fibronectin type III domains or other protein modules characterise the sub-families within this large group. Ig-like CAMs generally include type I transmembrane proteins with a large N-terminal domain, a transmembrane portion, and a cytoplasmic domain [1,14]. However, some CAMs are anchored to the membrane through a glycosylphosphatidylinositol (GPI) segment [15]. Some proteins demonstrate homophilic binding specificity, whereas others have heterophilic specificity, thus interacting with other Ig-like CAMs or with surface proteins [1,16]. Ig domains play critical roles in mediating homophilic and heterophilic interactions in *trans*, namely between CAMs on adjacent cells or CAMs localised to the extracellular environment, as well as in *cis*, i.e., with proteins located in the plasma membrane of the same cell [17,18]. The first Ig-like CAMs identified in the central nervous system (CNS) were neural cell adhesion molecule (NCAM) and L1, which, along with their isoforms, are recognised for their major functions in axon outgrowth and fasciculation, neuronal survival and migration, synapse formation, and synaptic plasticity [11,14,19]. Subsequent research led to the identification of additional sub-families, such as the nectins, MAM domain–containing GPI anchors, IGSF9, IGSF21, contactins, and IgLONs [11,20]. The IgLON subfamily, which is the focus of this review, includes five members sharing the presence of three C2 immunoglobulin domains in the N-terminus and a GPI-anchoring to the membrane (Figure 1). The first family members identified were Limbic System Associated Membrane Protein (LSAMP, initially named LAMP) [21], Opioid Binding Protein/Cell Adhesion Molecule Like (OPCML, also known as OBCAM) [22], and neurotrimin (NTM) [23]. Indeed, the name IgLON stands for Ig family containing LAMP, OBCAM, NTM. Subsequently, another family member was identified initially as KILON/neurotractin, and later re-named Neuronal Growth Regulator 1 (NEGR1) [24,25]. Lastly, antibodies against the IgLON member IgLON5 (IgLON Family Member 5) have been associated with autoimmune encephalitis [26]. Since the discovery of this family, its prominent roles in brain development, axon fasciculation, neurite extension, and synapse formation and maintenance have been identified [21,22,23,24,25]. Although the role in synapse regulation is well-established, further research is needed to characterise the molecular mechanisms underlying the functional outcomes [17,27,28,29,30]. A meaningful contribution to the comprehension of IgLONs’ physiological functions derived from the analysis of its molecular evolution, originating from an ancestor around the emergence of Arthropods [31]. Functional analysis allowed for the identification of common as well as specific sequences for interacting partner recognition, post-translational modifications, metalloproteinase cleavage sites, and signal transduction pathways [31].

Available evidence supports the involvement of CAMs in the onset of neuropsychiatric disorders. In Alzheimer’s disease, a role for CAMs has been suggested by genome-wide association studies (GWAS) as well as by the identification of altered levels in diseased brains [32,33]. Molecules belonging to several CAM families have been implicated in pathological manifestations through different mechanisms, involving amyloid-β metabolism, cell plasticity, and neuroinflammation [33,34]. The detection of an increase in the enzymes involved in GPI-anchoring in Alzheimer’s disease brains provided further support to a relevant role of these sub-families [35]. Moreover, genetic studies have associated CAMs with a number of neuropsychiatric disorders, including CRASH syndrome, MASA syndrome, X-linked mental retardation, intellectual disability, autism spectrum disorder, schizophrenia, addiction, and bipolar disorder [15,19]. The roles of CAMs in axon growth, guidance, and fasciculation; in target recognition; and in synapse formation and maintenance might contribute to the circuit alterations characterising neuropsychiatric disorders. The main proposed mechanisms include the ability to target the formation of specific circuitries responsible for specialised brain functions through localised expression. Additionally, CAMs may impact the occurrence of neuropsychiatric diseases by modifying the balance between excitatory and inhibitory signals, thereby affecting local neural circuit activity. This influence extends to the modulation of neuromodulatory systems like monoaminergic circuits and the alteration of synaptic strength and composition [15]. Focussing more specifically on Ig-like CAMs, mutations in the genes belonging to the best-studied NCAM and L1 families, as well as changes in their expression patterns or post-translational modifications, have been associated with psychiatric and neurodegenerative disorders, including the L1 syndrome, Alzheimer’s disease, schizophrenia, and bipolar disorder [36]. When further restricting the focus to GPI-anchored sub-families, their role as functional receptors controlling neurite outgrowth, synapse formation, synapse plasticity, and learning behaviours has been implicated in the pathophysiological mechanisms associated with neurodegenerative and psychiatric disorders [17]. Since a wealth of data has been recently accumulating to implicate the IgLONs, the aim of the present review is to analyse and discuss the findings indicating possible connections between this family and neurological and psychiatric disorders. 

## 2. IgLON5 (IgLON Family Member 5)

Anti-IgLON5 disease (ORPHA:420789) is a rare disease of the CNS. Since its discovery in 2014, 60 cases have been reported worldwide [37,38,39]. The clinical manifestation of the disease is highly heterogeneous, encompassing unique sleep and movement disorders, bulbar dysfunction, and cognitive impairment [26]. Disease onset is around the age of 60 years with progressive symptoms that can culminate in life-threatening respiratory problems [37]. Post-mortem analysis has revealed a tauopathy restricted to neurons in the brainstem, tegmentum, hypothalamus, and hippocampus [40]. The mortality rate associated with the anti-IgLON5 syndrome is considerably high, contributing to an overall mortality rate of 34%, with no discernible link between mortality and treatment response [38]. While respiratory complications are the primary cause of death, the additional neurological symptoms (cognitive decline, sleep disturbances) significantly diminish the quality of life for patients and their families.

**Figure 2:**
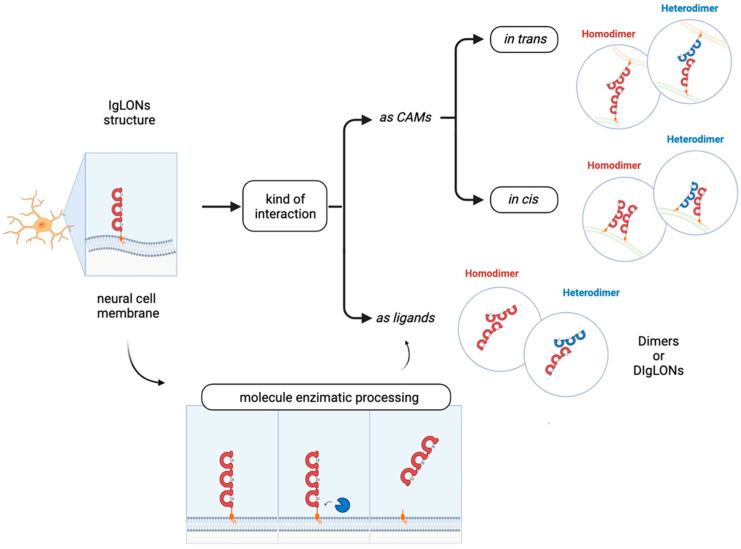
IgLONs can form homo- and hetero-dimers to interact in *trans* (in different cells) or in *cis* (in the same cell). They can be cleaved from the membrane by metalloproteases to form soluble interacting proteins.

The role of the IgLON5 protein and the underlying mechanism of disease associated with anti-IgLON5 antibodies remain poorly understood. IgLON5 is widely expressed in the CNS, with the highest levels reached in the olfactory bulb, cortical plate, and hippocampus in mice and humans. Comprising a chain of 336 amino acids, IgLON5 shares significant structural resemblance with other proteins such as OPCML (50% similarity), NTM (48–49% similarity), LSAMP (46–47% similarity), and NEGR1 (41% similarity) (Figure 1). IgLON5 is involved in forming both homomeric and heteromeric interactions with other IgLON family members [41] (Figure 2). However, the functional implications stemming from these interactions remain poorly characterised.

The detection of anti-IgLON5 antibodies is crucial for the diagnosing of anti-IgLON5 disease. These antibodies are detectable in both serum and cerebrospinal fluid (CSF), typically coinciding with the initial diagnosis timeframe. The question of whether anti-IgLON5 antibodies directly cause neuronal dysfunction and degeneration or instead appear as a consequence of the neurodegenerative process remain unresolved. Among anti-IgLON5 antibodies, the non-complement fixing IgG4 subclass predominates over IgG1. In vitro experiments have shown that IgG4 antibodies lead to the internalization of IgLON5 in cultures of hippocampal neurons [42]. Upon exposure to anti-IgLON5 antibodies, cultured rat or human neurons showed increased neurodegenerative features such as neuronal blebbing and fragmentation [43,44]. Prolonged exposure to anti-IgLON5 IgG prompts tau hyperphosphorylation and cellular death [44].

Similar to Alzheimer’s disease, the tau filaments evident in individuals with anti-IgLON5 syndrome encompass both 3R-tau and 4R-tau isoforms. However, this accumulation manifests in a distinct spatial pattern, indicative of anti-IgLON5 disease constituting a novel form of tauopathy. Recent autoptic studies have described anti-IgLON5 cases lacking hyperphosphorylated tau deposits [45,46]. These findings raise the possibility that tauopathy might arise subsequently in the disease progression, evolving as a prolonged outcome of the antibody-related effects. Thus, earlier events still depending on anti-IgLON5 antibodies may play a role in the aetiology of the disease, in addition to the proposition that a genetic predisposition to autoimmunity might exert influence, exemplified by the robust correlation with the exceptionally rare HLA-DRB1*1001 and HLA-DQB1*0501 alleles [40]. 

**Figure 3:**
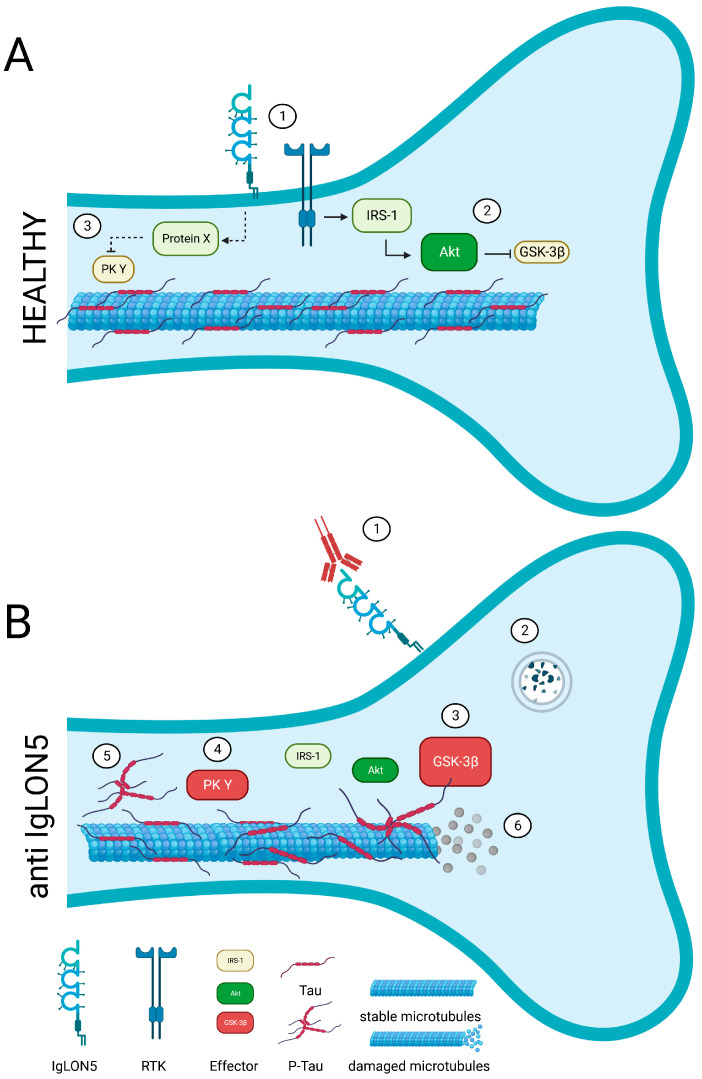
A potential link between IgLON5 and the aggregation of hyperphosphorylated-Tau. (**A**) In healthy conditions, IgLON5 may interact with and regulate a yet to be identified receptor tyrosine kinase (RTK, 1). A signalling cascade involving such RTK, insulin receptor substrate 1 (IRS-1), and AKT (2) prevents Tau phosphorylation by GSK-3β (3). A different RTK-triggered pathway may control Tau phosphorylation (4) and assure microtubule stability. (**B**) In patients, the anti-IgLON5 antibodies sequester IgLON5 (1) and trigger the degradation of IgLON5 together with the RTK, potentially (2). As such, RTK signalling pathways are altered (3–4). This may result in pathological tau phosphorylation and accumulation (5) and, eventually, neurotoxic microtubule disassembly (6).

IgLONs interact and regulate receptor tyrosine kinase (RTK) trafficking and, eventually, their signalling impinging on ERK1/2 and AKT phosphorylation [28,29]. Anti-IgLON5 disease is characterised by the aggregation of hyperphosphorylated-Tau, which is phosphorylated by a variety of serine/threonine protein kinases [47]; GSK-3𝛽 is the main kinase responsible for Tau phosphorylation and precipitation [48]. An intricate signalling cascade, encompassing RTKs, insulin receptor substrate 1 (IRS-1), and AKT, has been identified as a key mechanism in curbing Tau phosphorylation by GSK-3𝛽 [49]. Hence, the hypothesis that IgLON5 may interact with and regulate a yet to be identified RTK is exceptionally intriguing (Figure 3). Recent in vitro findings put forth the proposition that antibodies against anti-IgLON5 could potentially interfere with the IgLON5 interactome [50]. It is plausible that these anti-IgLON5 antibodies might impede the IgLON5-RTK interaction, thereby exerting an influence on RTK signalling. This sequence of events could consequently trigger aberrant Tau phosphorylation and accumulation, ultimately culminating in neuronal dysfunction (Figure 3).

In order to pave the way for effective therapeutic interventions geared towards individuals afflicted with IgLON5 deficiency disease, a comprehensive grasp of both the normal and pathological roles of IgLON5, alongside the associated signalling cascade, becomes essential.

## 3. NEGR1 (Neuronal Growth Regulator 1)

**Figure 4:**
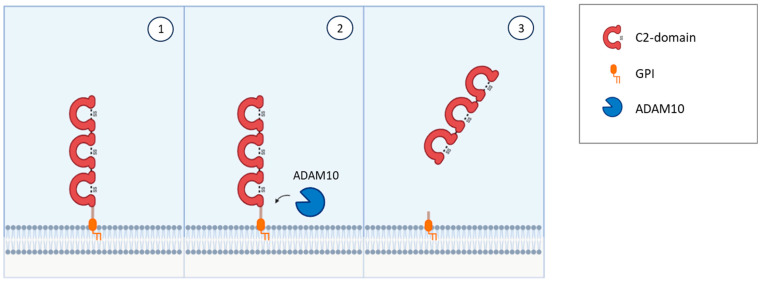
The metalloprotease ADAM10 can cleave IgLONs to generate soluble proteins able to bind RTKs and start intracellular signal transduction.

NEGR1 was first isolated and identified as a member of the IgLON family in 1999 in rats [24] and in chicken [25] as a 46–50 kDa protein; it presents the common class features of three amino-terminal C2 domains, six glycosylation sites, and GPI anchoring [24,25,51,52] (Figure 1). Homo- and heterodimerization with other IgLONs in *cis* and in *trans* across the synaptic cleft were recently thoroughly investigated [41,53] (Figure 2). Its expression in the brain is widespread, with high levels in the olfactory bulb, cerebral cortex, diencephalon, hippocampus, hypothalamus, and cerebellum [54,55,56,57], in a developmentally-regulated fashion [29,30,58,59]. NEGR1 is able to modulate neurite extension, either upon up- or down-regulation, suggesting that precise regulation is critical for correct functioning and promoting outgrowth in response to CNS injury [28,58,60]. Likewise, NEGR1 was identified as a crucial factor in determining the number of synapses in hippocampal neurons in opposite directions depending on environmental conditions [61], whereas knock-out mice showed impaired axon growth [59]. In cortical cultures, NEGR1 silencing decreased neurites’ total length and number. The reduced basal dendrite arborization induced by NEGR1 downregulation was also demonstrated in vivo in the somatosensory cortex of mice exposed to miRNA-transfection in utero [62]. Ectodomain shedding through the activity of metalloproteinases is a critical mechanism for IgLONs function (Figure 4). 

**Figure 5:**
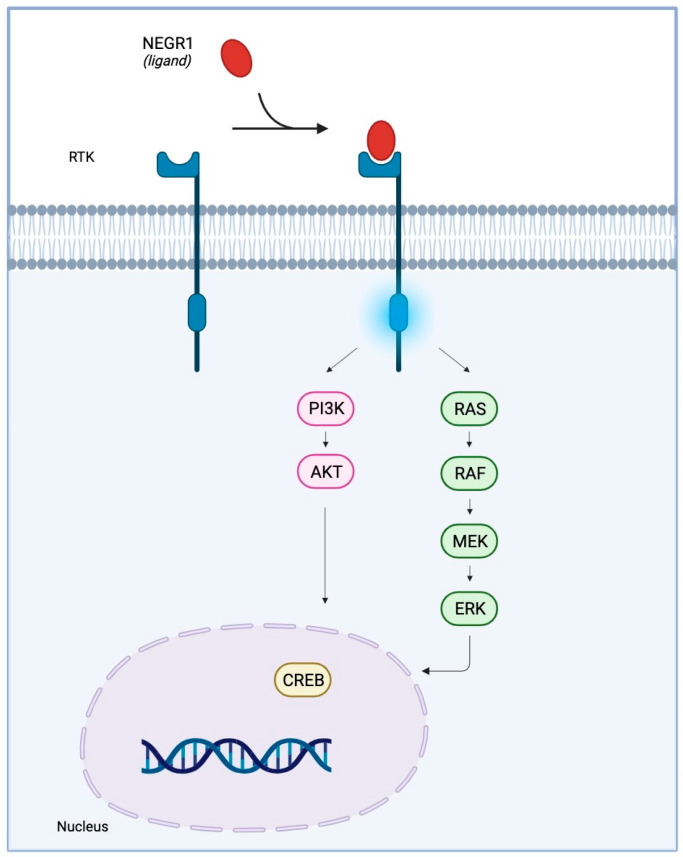
Soluble NEGR1 generated by shedding binds FGFR2 and activates intracellular signal transduction pathways.

In particular, the Disintegrin and Metalloproteinase Domain-Containing Proteins (ADAM) were demonstrated to be responsible for shedding NEGR1 and other IgLONs from the plasma membrane with a direct impact on neurite outgrowth in cultured cortical neurons [60]. Further research demonstrated that the metalloprotease ADAM10 is specifically involved in NEGR1 shedding and that the generation of soluble NEGR1 was able to regulate neuronal morphology by binding to the FGFR2 receptor and activating its intracellular pathway [28]. Among the latter, a prominent role is exerted by ERK and AKT pathways, which decrease FGFR2 degradation from the plasma membrane [28,29] (Figure 5). NEGR1’s impact on dendritic spine densities of pyramidal neurons has also been reproduced in vivo in the mouse somatosensory cortex [29]. Moreover, a role in hippocampal neurogenesis and long term potentiation are supported by the disruption of these functions in NEGR1 knock-out mice [63]. NEGR1 knock-out mice showed altered serotonergic and dopaminergic neurotransmission [64] and subtle alterations in social behaviour and in learning, as well as increased susceptibility to pentylenetetrazol-induced seizures [59]. In contrast with previous findings reporting no changes in anxiety [59], Noh et al. [63] showed that NEGR1 knock-out mice showed increased anxiety- and depressive-like behaviours, proposing the involvement of lipocalin and Leukaemia Inhibitory Factor (LIF) Receptor in the mechanism of action. 

In 2009, a GWAS meta-analysis identified NEGR1 among genetic determinants of the variation in body mass index in adults and children, with a stronger influence on adiposity, which is likely to be mediated by its expression in the hypothalamic nuclei [65,66,67,68,69]. In line with these findings, modulation of NEGR1 in rodents affected body mass index and energy balance in complex, developmentally regulated ways [70,71]. Recently, NEGR1 has been indicated among the principal players in the genetic correlations between obesity and a number of psychiatric disorders, including major depression, schizophrenia, and anorexia nervosa [72].

Within psychiatric disorders, a statistically significant association with major depressive disorder has been discovered in recent meta-analyses of GWAS, with NEGR1 resulting among the strongest signals [73,74,75,76]. By integrating GWAS, brain expression quantitative trait loci data, and transcriptome-wide association studies, the association was confirmed and assigned the highest probability of causality; increased abundance of NEGR1 was significantly associated with an increased depression risk [77,78,79]. Differential regulation of transcription factor binding and the consequential altered expression has been proposed as a potential mechanism of action of the single-nucleotide polymorphisms (SNPs) associated with the increased risk of major depression [80]. Adding further evidence to the significance for depression, NEGR1 levels in the CSF of patients affected by major depression or bipolar disorder were significantly higher than in controls; this biomarker provided a major contribution to the identification of a biosignature, allowing for the correct patient group stratification [81]. In rodent models, treatment with antidepressant medications altered NEGR1 expression in brain regions [82,83], whereas genetic variants have been associated with antidepressant treatment response in obese depressed patients [84]. Supporting a possible involvement in the pathophysiology of schizophrenia, elevated levels of NEGR1 mRNA were detected in the dorsolateral prefrontal cortex of patients, [85], in agreement with previous findings in anterior pre-frontal cortex samples [86]. The association was also demonstrated at the genetic level in human patients [87]. Still within the realm of psychiatric disorders, altered NEGR1 DNA methylation has been discovered in patients affected by anorexia nervosa [88].

Evidence to imply potential NEGR1 connections with the aetiology of neurological and neurodegenerative disorders has been accumulating recently. The existence of an association between NEGR1 and Alzheimer’s disease has been investigated due to the frequent comorbidity with major depression. Ni et al. [89] reported a significant genetic association with Alzheimer’s disease in Han Chinese patients, as well as altered expression in rodent models of the disease. These findings were expanded by a study identifying statistically significant associations with the protective efficacy of SNPs located in the NEGR1 gene to mediate the protective role of educational attainment in Alzheimer’s disease [90]. In healthy subjects’ brains, NEGR1 has been associated with white matter integrity measured as fractional anisotropy. This finding is highly suggestive, since altered white matter integrity characterises several neurodegenerative diseases, comprising Alzheimer’s disease [91,92,93]. In synucleinopathies, including Parkinson’s disease, NEGR1 has been indicated as a potential CSF biomarker in mass spectrometry studies, thus implicating relevance for diagnostic and therapeutic applications [94]. In line with these findings, NEGR1 levels in plasma were downregulated and in correlation with cognitive clinical scores and motor abilities in Parkinson’s disease patients [95]. An association between NEGR1 and transmissible spongiform encephalopathies, fatal neurodegenerative diseases characterised by infectious proteins, has been discovered in rodent models in which substantially decreased levels of NEGR1 have been demonstrated. Interestingly, NEGR1 was the only glycosylated biomarker among those identified [96]. A plausible role for NEGR1 in the neurodegenerative disorder Huntington’s disease has been reported by Kaltenbach et al. [97], who showed that the mutated form of huntingtin interacts with NEGR1 and that the latter is able to modify a neurodegeneration phenotype induced by treatment with a huntingtin fragment in *Drosophila*. NEGR1 is able to interact with Niemann–Pick disease type C2 protein, increase its stability, and thus modulate cholesterol accumulation and influence its homeostasis. This effect has been implicated in Niemann–Pick disease type C, an autosomal recessive disorder characterised by cholesterol accumulation leading to dementia [98]. 

Outside the realm of neuropsychiatric disorders, NEGR1 has been implicated in other diseases whose mechanisms may have a bearing on cognitive and intellectual disabilities that often denote neurodegenerative disorders. A possible relevance of NEGR1 in autism is supported by the observation that NEGR1 downregulation in mice reproduces brain development abnormalities resembling the cortical disorganization of neurons and abnormalities in dendritic spines characterising autism spectrum disorder features. In addition, NEGR1 and FGFR2 downregulation alters social skill-related behaviours similarly to autism spectrum disorder, in reducing ultrasound vocalizations in pups, causing sensory deficits in a hot plate test in newborns, and lowering social interactions in adults [29]. Furthermore, NEGR1 has been identified as a candidate susceptibility gene in human genetic studies [99]. An association of NEGR1 with dyslexia emerged in a copy number variation analysis in Indian families. These results suggest a potential role for NEGR1 in this neurogenetic disorder, possibly by influencing the production of dendritic postsynaptic spines in mature neurons [100]. Partial deletion of chromosome 1p31.1, only involving the NEGR1 gene, has been observed in two siblings affected by attention deficit hyperactivity disorder and language impairments, but not affected by dyslexia [101]. Another patient affected by microdeletion 1p31, including NEGR1, showed moderate intellectual disability [102]. Language impairments have been reported in a patient with an interstitial 1p31.1p31.3 deletion affecting several genes including NEGR1 [103]. A meta-analysis approach on gene expression data in multiple sclerosis patients’ tissues showed a significant increase in NEGR1 levels, which was interpreted as a leading cause of impaired synaptogenesis [104]. Additional support to NEGR1 contribution to this disorder derived from a study uncovering a potential impact in the comorbidity with major depression in multiple sclerosis patients [105]. 

## 4. OPCML (Opioid Binding Protein/Cell Adhesion Molecule Like)

The IgLON member OPCML (referred also as IgLON1 or OBCAM) binds opioid receptors after shedding from the plasmatic membrane, where it functions as a CAM [53,106] (Figure 2). The OPCML gene, comprising seven exons and spanning approximately 600 Kb, is mapped to human chromosome 11, which also hosts NCAM and other brain-expressed CAMs [107]. As a result of alternative splicing, four different transcripts have been identified [108]. Originally, OPCML was purified from rat brains and presumed to be an opioid binding protein [109], and then later recognised as a member of the IgLON subgroup on the basis of its cDNA sequence [22]. In general, the primary sequence of the protein is very highly conserved between species [22]. OPCML consists of 345 amino acids, with a molecular mass of 38 kDa, harbouring six glycosylation sites at Asn44, Asn70, Asn140, Asn285, Asn293, and Asn306 [22]. Depending on the grade of glycosylation or the presence of other post-translational modifications, the molecular weight of the protein can vary [51,110]. The structure of the OPCML ectodomain is characterised by the presence of three C2 domains [22,53] and the protein is anchored to the plasma membrane through a GPI site [17,53](Figure 1). Therefore, intracellular signalling can be induced by interacting in *cis* with other transmembrane proteins and through the formation of homo- or heterodimers with other IgLONs [111]. The ability of OPCML to act independently as an opioid receptor has been debated, reaching the conclusion that its playing an accessory role seems more likely, based on primary structure similarity to IgLONs instead of G-protein coupled receptors [22,107]. OPCML transcripts have been widely detected in brain and non-brain tissues in adult and foetal animal models and humans, with brain-specific enrichment of the v2 isoform [108,112]. In line with its high expression in neurons, OPCML takes part in cell adhesion and cell–cell recognition by interacting with and modulating molecules that can promote or inhibit growth, regulating synapse number, synaptogenesis, and plasticity [111,113]. 

Polymorphisms in the OPCML gene have been linked to schizophrenia in GWAS studies in European patients and the association was later confirmed in a Thai population [114,115,116]. In a genetic study on OPCML SNPs conducted in a Han Chinese population, the association was confirmed again and reduced hippocampal expression was found in risk-allele carriers [117]. However, expression levels were not altered in the prefrontal cortex of adult patients with schizophrenia, suggesting that the control of transcription in the adult brain may not represent a prominent mechanism of the association [118]. More recently, a large meta-analysis combining data from several repositories highlighted another OPCML-associated SNP (rs2917569) with a high grade of association [119]. Although the role of OPCML in the pathophysiology of schizophrenia requires clarification, its crucial function in neurite outgrowth and spine maturation has been implicated through Eph-Cofilin signalling and F-actin polymerization [117]. Still within psychiatric disorders, a major depression study in two independent Dutch populations highlighted a significant linkage with two SNPs on chromosome 11q25 located in the intronic region 1 of the OPCML gene. The finding has been interpreted as due to the relevance of endogenous opioid neurotransmission in depressed patients [120]. Furthermore, a GWAS on anorexia nervosa detected a signal for an intronic SNP in the OPCML gene which was associated with disease risk across all discovery and replication cohorts, although it did not reach genome-wide significance [121]. A case report of two brothers with disruption in the chromosomal region encompassing OPCML and NTM suggested possible links with autistic symptoms [122]. 

Within neurodegenerative diseases, OPCML has been implicated in Alzheimer’s disease in a GWAS carried out in patients affected by the late-onset form [123]. In line with these findings, altered OPCML mRNA levels were identified in the hippocampus of two mouse models of disease [124]. In addition, reduced OPCML glycosylation in Alzheimer’s disease brains, together with other CAMs, was interpreted as a driver of altered cell adhesion and synaptic function in the disorder [125]. 

## 5. LSAMP (Limbic System Associated Membrane Protein)

The limbic system associated membrane protein (LAMP or LSAMP, as indicated in more recent works to distinguish it from the lysosomal associated membrane protein) is an IgLON family member first identified as specifically characterising neurons belonging to the rat limbic system or to areas receiving direct projections from there [21]. Subsequent purification and cloning revealed that LSAMP is an integral membrane protein with a molecular mass of 64–68 KDa containing three internal repeats typical of the Ig-like domain, with conserved pairs of cysteine residues, together with eight putative glycosylation sites [126,127,128] (Figure 1). Like the other IgLONs, it is a membrane protein containing a GPI anchor, which can be released from intact membranes by the action of phosphatidyl inositol-specific phospholipase C [129]. LSAMP nucleotidic and aminoacidic sequences present high sequence identity with those of OPCML and NTM, suggesting similar roles for these proteins [127]. In neurons, most protein expression is post-synaptic, located on neuronal somata and dendrites, while glial cells are not immunoreactive [126]. In the adult rat, LSAMP is expressed in regions related to the limbic system, like the hippocampus, amygdala, perirhinal cortex, anterior and lateral thalamic nuclei, preoptic area of the hypothalamus, septum, nucleus of the solitary tract, and lamina II of the dorsal horn [21,126,128,130]. The specificity of this protein for neurons belonging to the same functional system suggest its possible involvement in brain regionalization during development. Indeed, during development LSAMP mRNA is expressed in the same limbic regions as in the adult, but in a temporal fashion that relates with the time of formation of limbic axonal pathways [128,130,131]. In addition, ultramicroscopic analysis revealed that in embryonic tissue the localization of LSAMP is mainly located in the growth cone of developing axons [126,131]. The axonal staining disappeared during the second post-natal week of development, indicating a role for LSAMP in axonal growth and limbic pathway formation [130,131].

The in vivo administration of anti-LSAMP antibodies altered the formation of mossy fibres projection to pyramidal neurons during hippocampal development, suggesting that LSAMP may serve as a recognition molecule for the proper genesis of limbic connections [127]. LSAMP’s crucial role in establishing limbic connections as well as limbic neural fate was further highlighted by experiments where the connections between neurons belonging to different parts of the brain were manipulated or disrupted [130,132,133,134]. These data suggest that the commitment to limbic fate happens early in development, and that LSAMP represents an early marker of limbic neurons determination. Thus, LSAMP acts as an attractive molecule, guiding the sprouting of limbic axons, and behaving as a repulsive signal for the growing of non-limbic axons [135]. The heterophilic binding both in *cis* and in *trans* with other IgLON members mediated by the different IgG domains has been identified as a critical mechanism of action [25,111,136,137,138,139,140] (Figure 2). Another important mechanism of regulation in the development of axonal growth cones involves the molecular shedding of the IgLON ectodomain from the membrane by metalloproteases, with a prominent role played by ADAM10 [60,141] (Figure 4). 

In pre-clinical models of disease, evidence of alterations in the molecular expression of LSAMP is available, suggestive of implication in the neurobiological underpinnings of psychiatric disorders. Increased LSAMP expression was found in rats with low exploratory activity in the elevated plus maze test, suggesting a correlation with anxiety [142,143]. Further evidence derives from LSAMP knock-out mice, which demonstrated a heightened responsiveness to novelty in behavioural tests as well as a maladaptive response to environmental stressors and altered interaction with the serotoninergic system, associated with increased sensitivity to psychostimulants [142,144,145,146,147,148]. Moreover, LSAMP deletion impacts synaptogenesis and synaptic plasticity, hippocampus formation, and spatial memory [57,59,149]. In human genetic studies, SNPs in the LSAMP gene have been associated with male suicide [150] when it is well established that the most important risk factor for suicide is depression [151]. Indeed, a significant association with major depression and panic disorder has been detected [152]. In addition, altered LSAMP levels were observed in the dorsolateral prefrontal cortex of schizophrenic patients, albeit with contrasting findings in different patient cohorts [85,153]. In line with these results, significant allelic and haplotypic associations have been reported between SNPs in the LSAMP gene and schizophrenia [154]. 

Little is known about LSAMP involvement in neurodegenerative diseases. A study aiming at identifying biomarkers for Lewy bodies dementia, Alzheimer’s disease, and Parkinson’s disease in patients’ CSF recognized LSAMP as a novel non-specific biomarker for neurodegeneration due to its significantly increased expression in all three diseases with respect to controls [155]. A similar study, where the relative expression of N-glycosylated proteins in Alzheimer’s disease was analysed through mass spectrometry, showed that LSAMP negatively correlated with the disease, suggesting that decreased post-translational modification can occur [125]. However, neither report provided clues about the molecular mechanisms of LSAMP involvement in neurodegeneration, thus further studies are required.

## 6. NTM (Neurotrimin)

NTM was characterised in 1995 as a 65 kDa integral protein endowed with three C2 amino-terminal domains, seven glycosylation sites, GPI anchoring, and high sequence homology with other IgLONs [23,156] (Figure 1). Like them, NTM can bind other family members in *cis* and in *trans*, as well as form homodimers [41,53,111,136,137,157] (Figure 2). The dimers are essential for NTM function in the regulation of neurite outgrowth and cell adhesion to control neuronal connectivity and synaptogenesis [139,157,158,159,160]. Both membrane-bound and soluble forms are involved in outgrowth regulation, with sensitivity to metalloproteases, particularly ADAM10 [60,141,157,161] (Figure 4). NTM is expressed in the developing and adult brain, with high expression in the sensorimotor cortex, olfactory bulb, thalamus, hypothalamus, hippocampus, basal ganglia, and cerebellum [23,137,156]. Differently from LSAMP knock out mice, NTM deficient mice had no differences in anxiety, social interaction, or locomotor activity, only sharing a lower sensitivity to the locomotor stimulating effect of amphetamine, although to a lower extent, and displaying subtle deficits in cognitively challenging emotional learning tasks [162]. 

SNPs in the NTM gene have been associated with intelligence in a family-based low-density genome-wide study; these findings are noteworthy since lower cognitive function increases the risk of neuropsychiatric disorders [163]. Autism spectrum disorder patients harboured copy number variants encompassing the NTM gene and two patients with a chromosomal translocation disrupting the NTM gene displayed autistic symptoms [122,164]. Furthermore, a signal for association with NTM has been discovered for childhood aggressiveness in attention deficit hyperactivity disorder, although the signal did not reach statistical significance [165]. A GWAS in a genetically isolated population from the Netherland linked four SNPs placed within intron 1 of the NTM gene to late-onset Alzheimer’s disease [123]. 

## 7. Conclusions

**Figure 6:**
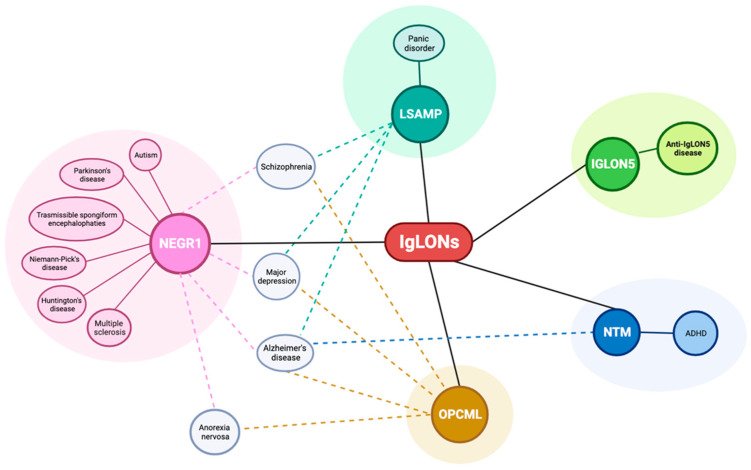
Associations between IgLONs and neuropsychiatric disorders.

A wealth of data is available linking IgLON with neurodegenerative, neurological, and psychiatric disorders (Figure 6); however, since most findings derive from genetic-association studies, the underlying mechanisms of action are poorly understood. 

For this family, the available evidence supports functions in nervous system development, neurite extension, synaptic formation and maintenance, blood-brain barrier structure, extracellular matrix protein recognition, and activation of signal transduction within cells able to elicit long-term responses. It is likely that the physiological role of IgLONs exerts a critical function in the pathophysiology of these disorders. However, the neurobiological underpinnings are yet to be investigated. We can speculate that the established function in synaptic plasticity and strength exerts a potential role in learning and memory formation, which can represent a common dysregulation impinging on the molecular underpinning of several disorders, with a prominent impact on neurodegenerative diseases. Dendritic spine pathology is an established hallmark of neurodegenerative diseases [166]. The accumulation of IgLONs in CSF samples gathered from patients affected by neurodegenerative disorders may reflect the spine loss. In addition, the discovery of the autoimmune response associated with anti-IgLON5 disease hints to a possible involvement of autoimmune responses as a contributing mechanism of action in other neurodegenerative and neurodevelopmental disorders associated with this family [167]. Accumulating evidence shows that IgLONs interact and regulate RTK trafficking and, eventually, their signalling impinging on ERK1/2 and Akt phosphorylation [28,29,168]. RTKs are tightly involved in the dynamics of the actin and tubulin cytoskeleton. An intriguing hypothesis is that IgLONs influence the signalling of RTKs and, consequently, cytoskeletal stability. Alteration in the cytoskeleton, such as the tau phosphorylation/accumulation observed in anti-IgLON5 disease, may eventually cause neuronal dysfunction and death.

As a caveat, it should be underlined that the existence of genetic associations does not necessarily imply a prominent role in the neurobiological underpinning of the disorder. In the same line, phenotypes associated with genetic deletions do not automatically imply a general role for the deleted gene in the pathology. Moreover, the partial overlap of disease association with IgLON family members supports the possible cross-disease alterations to be imputed to these CAMs. Further studies will elucidate the potential relevance of IgLON family members as future targets for therapeutic intervention or as biomarkers to aid diagnosis.

## Data Availability

Not applicable.
